# Postnatal Growth Restriction in Mice Alters Cardiac Protein Composition and Leads to Functional Impairment in Adulthood

**DOI:** 10.3390/ijms21249459

**Published:** 2020-12-12

**Authors:** Joseph R. Visker, Lawrence J. Dangott, Eric C. Leszczynski, David P. Ferguson

**Affiliations:** 1 Department of Kinesiology, Michigan State University, East Lansing, MI 48824, USA; joe.visker@utah.edu (J.R.V.); leszczy8@msu.edu (E.C.L.); 2 The Nora Eccles Harrison Cardiovascular Research and Training Institute, The University of Utah, Salt Lake City, UT 84112, USA; 3 Protein Chemistry Laboratory, Department of Biochemistry and Biophysics, Texas A&M University, College Station, TX 77843, USA; dangott@tamu.edu

**Keywords:** DOHaD, postnatal growth restriction, cardiac function, p57^kip2^, titin, collagen

## Abstract

Postnatal growth restriction (PGR) increases the risk for cardiovascular disease (CVD) in adulthood, yet there is minimal mechanistic rationale for the observed pathology. The purpose of this study was to identify proteomic differences in hearts of growth-restricted and unrestricted mice, and propose mechanisms related to impairment in adulthood. Friend leukemia virus B (FVB) mouse dams were fed a control (CON: 20% protein), or low-protein (LP: 8% protein) isocaloric diet 2 weeks before mating. LP dams produce 20% less milk, inducing growth restriction. At birth (postnatal; PN1), pups born to dams fed the CON diet were switched to LP dams (PGR group) or a different CON dam. At PN21, a sub-cohort of CON (*n* = 3 males; *n* = 3 females) and PGR (*n* = 3 males; *n* = 3 females) were euthanized and their proteome analyzed by two-dimensional differential in-gel electrophoresis (2D DIGE) and mass spectroscopy. Western blotting and silver nitrate staining confirmed 2D DIGE results. Littermates (CON: *n* = 4 males and *n* = 4 females; PGR: *n* = 4 males and *n* = 4 females) were weaned to the CON diet. At PN77, echocardiography measured cardiac function. At PN80, hearts were removed for western blotting to determine if differences persisted into adulthood. 2D DIGE and western blot confirmation indicated PGR had reductions in p57^kip2^, Titin (Ttn), and Collagen (Col). At PN77, PGR had impaired cardiac function as measured by echocardiography. At PN80, western blots of p57^kip2^ showed protein abundance recovered from PN21. PN80 silver staining of large molecular weight proteins (Ttn and Col) was reduced in PGR. PGR reduces cell cycle activity at PN21, which is recovered in adulthood. However, collagen fiber networks are altered into adulthood.

## 1. Introduction

Growth restriction caused by early life undernutrition increases the occurrence of non-communicable chronic disease in adulthood [1,2,3,4,5,6,7,8,9,10]. In particular, early life growth restriction increases the risk of cardiovascular disease (CVD) in adulthood by 47% [2,4,5,6,10,11]. CVD morbidity continues even if growth is recovered through a nourishing diet, demonstrating that nutritional insults during key developmental windows can program cardiac function into adulthood [2,5,6,7,12]. The clinical significance of the developmental programming of CVD is evident by the fact that over 7 million CV deaths occur each year in adults who have suffered growth restriction, with an associated annual healthcare cost of USD 42.5 billion [6].

While there is extensive evidence on CV pathology due to intrauterine growth restriction [13,14,15,16], there is far less on the dysfunction caused by postnatal growth restriction (PGR). From the evidence available, PGR in a mouse impairs cardiac function due to a greater amount of mononucleated cardiomyocytes [7,17], impaired calcium flux [17], stiffer ventricles leading to diastolic dysfunction [7], and increased incidence of cardiac arrhythmias [12] when compared to non-growth restricted mice.

Despite the above outlined CV pathology, there are limited data on the molecular mechanisms responsible for functional impairment, which encumbers the development of evidence-based therapies to mitigate cardiac impairment. Two-dimensional differential in-gel electrophoresis (2D DIGE) coupled with mass spectroscopy (MS) is a reliable technique to identify alterations in global protein abundance, and has been used previously for the proposal of mechanisms for chronic disease development [18,19]. As such, the present investigation used a nutritive model to induce PGR in mice [12,20] and analyzed a cohort of mice at postnatal day (PN) 21 by 2D DIGE and MS to identify differences in cardiac protein abundance immediately following the nutritional shortage. Afterwards, validated bioinformatics methods were utilized to construct a mechanistic pathway for which PGR influences cardiac development and function.

The remaining littermates were weaned to a fully nourished CON diet until adulthood, when echocardiography and Doppler analysis assessed cardiac function. Mice were then euthanized, and cardiac protein was evaluated with western blotting and silver nitrate staining to determine if the protein signatures identified at PN21 were recovered or persisted into adulthood.

## 2. Results

### 2.1. Growth

#### 2.1.1. PN1–21

**Figure 1:**
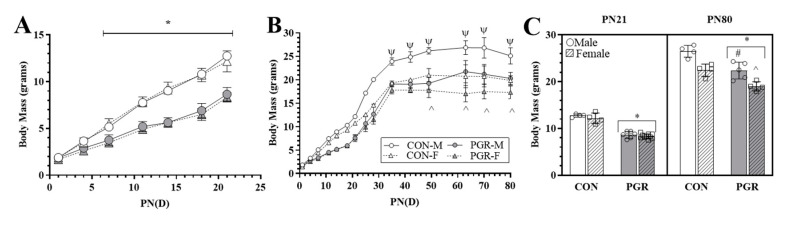
Effects of postnatal growth restriction (PGR) on growth: (**A**) Postnatal Day 1-21 (PN1–PN21) growth rate: PGR significantly (*; *p* < 0.001) reduced growth rate starting at PN7, with no sex effect, when compared with control (CON). (**B**) PN22–PN80 growth rate: PGR significantly (*p* < 0.001) reduced body mass at several time points (PN35, 42, 49, 63, and 70). The Control-Male (CON-M) mass was significantly (Ψ: *p* < 0.001) larger than all other groups. There was no significant (*p* = 0.967) difference between Control-Females (CON-F) and Postnatal Growth Restricted-Males (PGR-M). From PN49 to PN80, Postnatal Growth Restricted-Females (PGR-F) were significantly reduced compared to CON-F (^: *p* < 0.01). (**C**) Body mass (PN21/PN80): PN21-PGR (*n* = 16) mass was significantly less (*: *p* < 0.001) than for the CON group (*n* = 8), with no sex effect. PN80-PGR (*n* = 9) mass was significantly reduced (*: *p* < 0.001) compared to the CON group (*n* = 8). PGR-M were significantly less than CON-M (#: *p* < 0.001), and PGR-F were significantly less than CON-F (^: *p* < 0.01). PGR-M vs. CON-F was non-significant (*p* > 0.05).

PGR significantly (*p* < 0.001) decreased the growth rate from PN1–21 (PGR; males: 0.318 ± 0.017 g∙day^−1^ and females: 0.314 ± 0.016 g∙day^−1^) when compared with the CON group (males: 0.534 ± 0.013 g∙day^−1^ and females: 0.516 ± 0.022 g∙day^−1^), with no sex effect present (Figure 1A). PN21 body mass of PGR mice (males: 8.6 ± 0.731 g and females: 8.3 ± 0.518 g) was significantly less (*p* < 0.001) than for the CON mice (males: 12.7 ± 0.331 and females: 12.1 ± 1.132 g; Figure 1C), with no sex effect.

#### 2.1.2. PN22–80

PGR significantly (*p* = 0.005) decreased the growth rate from PN22–80 (PGR; males: 0.140 ± 0.036 g∙day^−1^ and females: 0.083 ± 0.026 g∙day^−1^) when compared with the CON growth rate (males: 0.138 ± 0.033 g∙day^−1^ and females: 0.113 ± 0.025 g∙day^−1^) (Figure 1B). The CON group had a significantly higher body mass compared to the PGR group after re-feeding that persisted over the course of the study (Figure 1C), resulting in the PGR mice having a decreased (*p* < 0.001) final body mass (PGR; males: 22.36 ± 1.79 g and females: 19.00 ± 0.98 g) when compared to the CON group (males: 26.50 ± 1.27 g and females: 22.45 ± 1.31 g). There was a significant sex effect (*p* < 0.001), with males being larger than females.

### 2.2. Heart Mass and Tibia Length

#### 2.2.1. PN21

**Figure 2:**
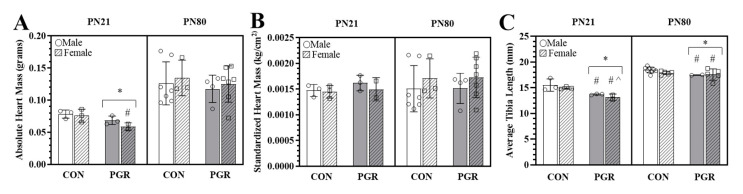
Effects of PGR on heart mass and tibia length: (**A**) absolute heart mass (PN21/PN80): The PN21-PGR group (*n* = 6) was significantly reduced compared to CON (*n* = 6); (*: *p* = 0.01). PGR-F were significantly less than CON-M (#: *p* = 0.05) but not less than CON-F (*p* = 0.09). PN80-PGR was not significantly (*p* > 0.05) different from the CON group, and no sex effect was present. (**B**) Standardized heart mass (PN21/PN80): no significant differences (*p* < 0.05). (**C**) Tibia length (PN21/PN80): PGR significantly reduced average tibia length compared to the CON group at PN21 (CON: *n* = 6, PGR: *n* = 6) and PN80 (CON: *n* = 12, PGR: *n* = 7); (*: *p* < 0.001), with no sex effect present at PN21. At PN80, a diet x sex interaction (#: *p* = 0.04) revealed the tibias of PGR-M and -F were smaller than CON-M but not CON-F (^: *p* < 0.01). Values are mean ± SD.

Absolute heart mass (Figure 2A) of the PGR group (males: 0.069 ± 0.006 g and females: 0.059 ± 0.005 g) was significantly (*p* = 0.014) less than for the CON group (males: 0.078 ± 0.006 g and females: 0.076 ± 0.010 g), with no sex effect present. When heart mass was standardized to body surface area (Figure 2B), no significant (*p* = 0.330) differences were found between PGR (males: 0.0016 ± 0.0001 kg/cm^2^ and females: 0.0014 ± 0.0002 kg/cm^2^) and CON (males: 0.0014 ± 0.0001 kg/cm^2^ and females: 0.0014 ± 0.0001 kg/cm^2^) groups.

PGR significantly (*p* = 0.002) reduced tibia lengths (PGR; males: 13.71 ± 0.11 mm; females: 13.15 ± 0.63 mm, Figure 2C) when compared to the CON group (males: 15.520 ± 1.12 mm and females: 14.97 ± 0.25 mm), with no sex effect present.

#### 2.2.2. PN80

At PN80, there were no significant (*p* = 0.991) differences in absolute heart mass (Figure 2A) between the PGR group (male: 0.117 ± 0.02 g and female: 0.125 ± 0.02 g) and CON group (male: 0.126 ± 0.03 g and female: 0.135 ± 0.03 g). When hearts were standardized to body surface area (Figure 2B), there were no significant (*p* = 0.953) differences between groups (PGR male: 0.0015 ± 0.0002 kg/cm^2^ and PGR female: 0.0017 ± 0.0003 kg/cm^2^; CON male: 0.0015 ± 0.0004 kg/cm^2^ and CON female: 0.0017 ± 0.0003 kg/cm^2^).

Tibias of the PGR group (male: 17.18 ± 0.28 mm; female: 17.67 ± 0.27 mm) were significantly smaller (*p* < 0.001, Figure 2C) than the CON group (male: 18.57 ± 0.5 mm; female: 18.15 ± 0.14 mm). Post hoc analysis revealed that the PGR males (*p* = 0.002) and females (*p* = 0.014) were only significantly different from the CON males, not CON females. The PGR males were smaller but not significantly (*p* = 0.05) when compared to CON females.

### 2.3. 2D DIGE and LC/MS/MS Analysis

A total of 2187 spots were detected in six CyDye-labeled gels. From this, two spots were determined to be significantly different in volume (*p* < 0.05) between CON and PGR and an additional three spots had lesser differences in spot volumes with a *p*-value of 0.05–0.075 (Table 1). All five spots were analyzed by nano-LC/MS/MS. Statistical analysis of the spot volumes of the two conditions (CON or PGR; no sex effect was present) revealed 12 possible proteins in the significant spot volumes. An additional 18 possible proteins were indicated in the other three spots and are reported as an avenue for future investigations. The 12 potential proteins from the spots with a *p* < 0.05 indicated reduced PGR protein abundance as compared to the CON group. The individual proteins identified by 2D DIGE and LC/MS/MS were associated with extracellular matrix organization/muscle contraction (21.5% lower abundance of Titin (Ttn), collagen type I α 1 chain, collagen type III α 1 chain, and collagen type IV α 5 chain), cell cycling/signal transduction (20% lower abundance of p57^kip2^, exportin 5, and Rho GTPase activating protein), and metabolism (25.42% lower abundance of peroxiredoxin 6, SAFB-like transcription modulator).

### 2.4. Western Blotting of p57^kip2^

PGR impairs cardiomyocyte nucleation, leading to functional impairments in adulthood [17], therefore, a cell cycle regulator, p57^kip2^, was classified as a protein of interest based on existing literature and significant 2D DIGE results [15,17,21,22,23,24].

#### 2.4.1. PN21

**Figure 3:**
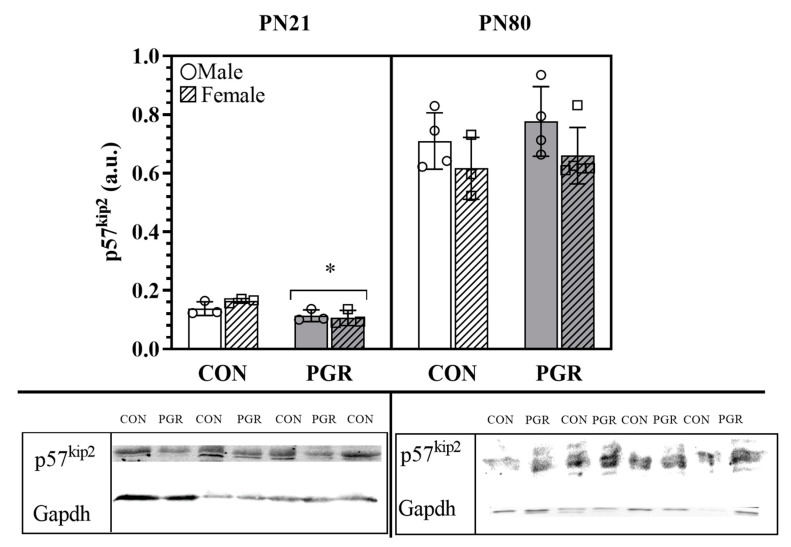
Western blotting protein abundance of p57^kip2^: PN21; p57^kip2^ PGR (*n* = 6) abundance was significantly less than for CON (*n* = 6); (*; *p* = 0.007). At PN80, no significant differences were seen, as abundance is recovered (*p* > 0.05; CON: *n* = 7, PGR: *n* = 9). Values are mean ± SD. The blots below the bar graphs are contiguous, representative bands selected from the gels.

In agreement with 2D DIGE and MS, the relative protein abundance of p57^kip2^ was significantly (*p* = 0.007) lower in the PGR group (0.110 ± 0.021 arbitrary units (a.u.)) compared to the CON group (0.151 ± 0.021 a.u., Figure 3).

#### 2.4.2. PN80

The relative protein abundance of p57^kip2^ in adulthood was recovered (Figure 3) and showed no significant differences (*p* = 0.363) between the samples of PGR (0.702 ± 0.121 a.u.) and CON (0.670 ± 0.104 a.u.) hearts.

### 2.5. Silver Nitrate Staining

Based on the existing literature and significant 2D DIGE results, Ttn and Collagen (Col) proteins were classified as proteins of interest, as growth restriction also alters cardiac morphology [22,23], stiffens coronary arteries [15,25], rearranges the extracellular matrix [26], and impairs collagen deposition [27].

#### 2.5.1. PN21

At PN21, Ttn abundance was reduced but not significant (*p* = 0.065, Figure 4A) in PGR (2015 ± 484.6 a.u.) vs. CON (4549 ± 3734 a.u.).

**Figure 4:**
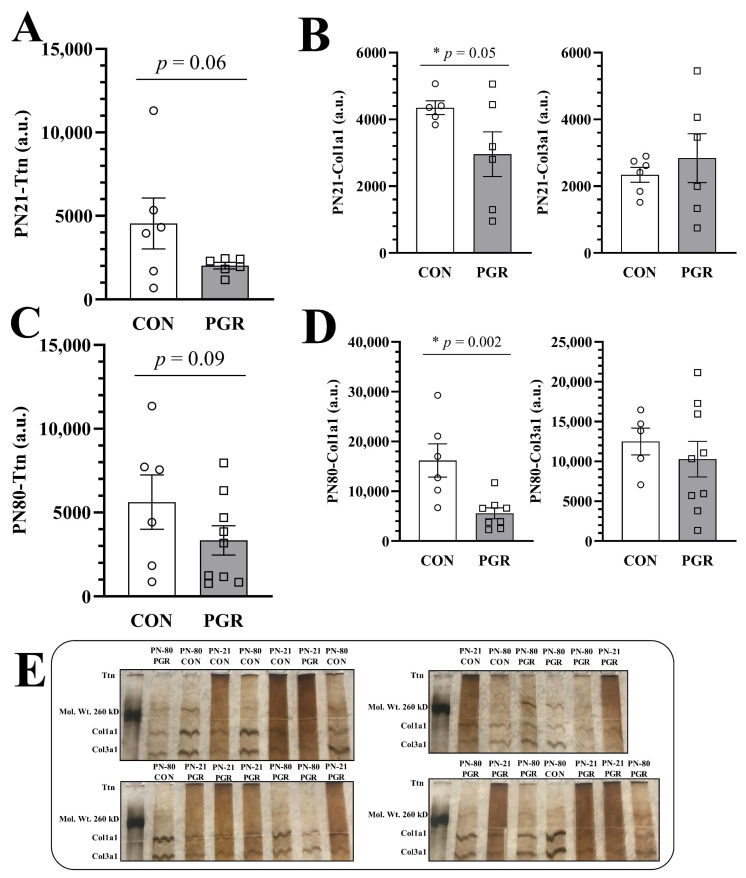
Silver nitrate stain of high molecular weight proteins in CON and PGR mice. (**A**) PN21-PGR-Ttn abundance (*n* = 6) was less than CON abundance (*n* = 6), but not significantly (*p* = 0.06). (**B**) In accordance with 2D-DIGE, PN21-PGR-Col1a1 abundance (*n* = 6) was significantly reduced compared to CON abundance (*n* = 5), (*p* = 0.05), however, there was no significant difference seen in PN21-Col3a1 abundance. (**C**) No significant differences (CON: *n* = 6, PGR: *n* = 9). (**D**) PGR-Col1a1 abundance (*n* = 8) continued to be less than CON abundance (*n* = 5) at PN80 (*p* = 0.002). There were no significant differences seen in PN80-Col3a1 abundance. (**E**) Silver nitrate-stained gels; CON and PGR cardiac protein samples (2 µg) were randomly loaded onto four gels at PN21 and PN80. As no sex differences were seen through two-dimensional differential in-gel electrophoresis (2D DIGE), males and females have been combined into their respective diet groups (CON or PGR). The stain is a contiguous, representative image of CON vs. PGR. Values are mean ± SEM.

PGR-Col1a1 abundance (2956 ± 1641 a.u.) was significantly reduced (*p* = 0.050, Figure 4B) compared to CON (4349 ± 461.0 a.u.). However, there were no significant differences (*p* = 0.263) in PN21-Col3a1 abundance (CON: 2340 ± 544.0 a.u. vs. PGR: 2841 ± 1794 a.u.).

#### 2.5.2. PN80

At PN80, PGR-Ttn abundance (3333 ± 2604 a.u.) was less than for CON (5623 ± 3986 a.u.), but not significantly (*p* = 0.099, Figure 4C).

PGR-Col1a1 abundance (5582 ± 3129 a.u.) was not recovered and remained significantly reduced (*p* = 0.002, Figure 4D) compared to CON (16,172 ± 8156 a.u.). Again, there were no significant differences (*p* = 0.257) between PN80-Col3a1 abundance of CON (12,501 ± 3759 a.u.) and PGR (10,288 ± 6722 a.u.). Silver nitrate-stained gels of CON vs. PGR can be viewed in Figure 4E.

### 2.6. Pathway Analysis

**Figure 5:**
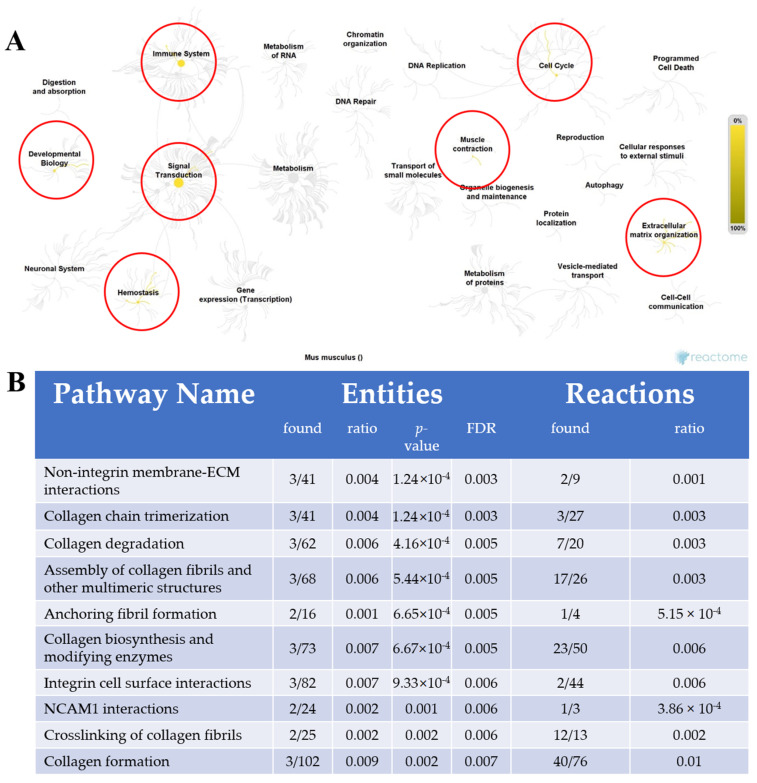
Proposed pathways for cardiac impairment caused by PGR. (**A**) Pathway network generated by REACTOME (yellow lines: 42 pathways involved). The most notable pathway hubs (red circles) were extracellular matrix organization (ECM), cell cycle, muscle contraction, signal transduction, developmental biology, immune system, and hemostasis. (**B**) Representative image generated by REACTOME of the 10 most relevant pathways sorted by *p*-value (<0.05), shown in a table. FDR: false discovery rate.

Using REACTOME software, 42 pathways were associated with the five proteins from 2D DIGE (p57^kip2^, Ttn, Col1a1, Col3a1). The identified hubs (a combination of interactors, entities, and reactions) included extracellular matrix organization, cell cycle, muscle contraction, signal transduction, developmental biology, immune system, and hemostasis (Figure 5).

### 2.7. Interaction Network

**Figure 6:**
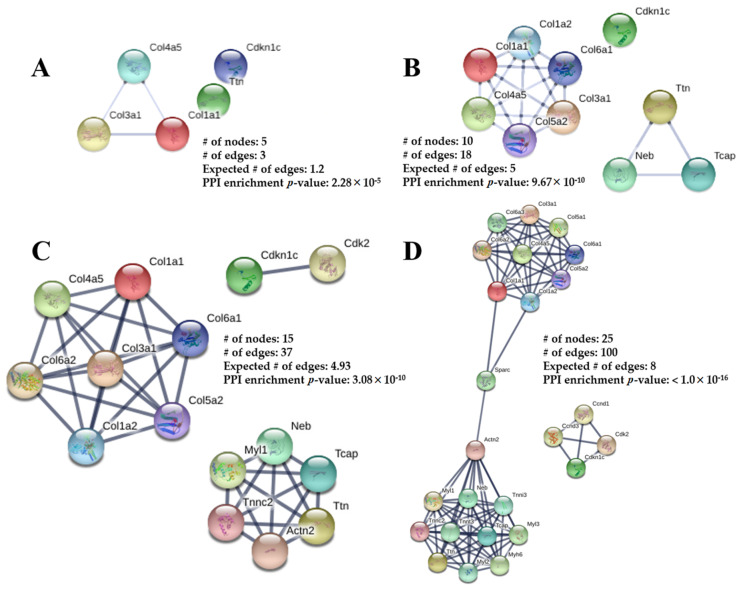
The Protein-Protein Interaction (PPI) networks constructed by the STRING database for the differentially abundant proteins from PGR and CON mouse hearts. The networks consist of the total number of nodes (proteins involved), number of edges (PPI connections amongst nodes), and a PPI enrichment *p*-value. The *expected number of edges* provides how many edges are anticipated if the nodes were selected at random. (**A**) No interactors. (**B**) Five predicted protein interactors added. (**C**) Ten predicted protein interactors added. (**D**) Twenty predicted protein interactors added. The spheres represent the proteins and the line thickness represents the PPI-associated degree of evidence (combination of gene fusion, gene neighborhood, gene co-occurrence, experimental evidence, text-mining evidence, database evidence, and co-expression evidence). The information inside of the circle describes the protein structure and the color of the nodes is for visual representation.

**Figure 7:**
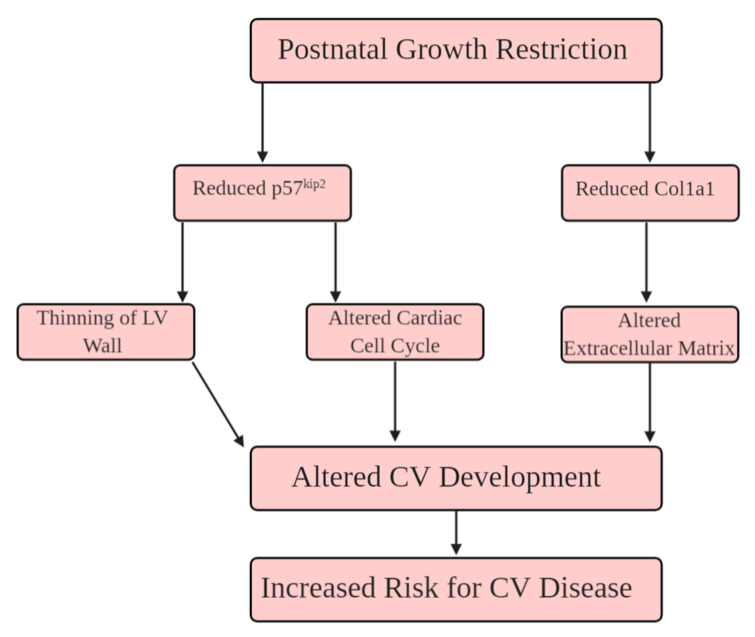
Hypothesized schema of how PGR alters extracellular matrix organization, and the cell cycle. (1) p57^kip2^ is reduced during PN21, leading to impaired cardiomyocyte maturation, and thinning of the left ventricular (LV) wall. (2) Reduced collagen fiber networks impair the extracellular matrix into adulthood. The convergence of these proteomic alterations in the PGR heart increase the risk for cardiovascular disease (CVD) in adulthood.

Using STRING software, four networks are presented in Figure 6A–D. The network in Figure 6A contains the five confirmed 2D DIGE proteins (total nodes/proteins involved) without any software-predicted interactions. Col1a1, Col3a1, and Col4a5 represent three edges (protein-protein interactions: PPIs between nodes), with an associated PPI enrichment *p*-value of 2.28 × 10^−5^. Figure 6B–D have been enriched with software-predicted interactors, meaning protein interactions are biologically connected or react with additional proteins not identified from 2D DIGE. The other 2D DIGE proteins, such as titin (Ttn) and p57^kip2^ (Cdkn1c), can predictably interact with other proteins, such as Neb, Tcap, and Cdk2. The *p*-values for Figure 6B–D are 9.67 × 10^−10^, 3.08 × 10^−10^, and <1.0 × 10^−16^, respectively, indicating the enriched networks are not random and the observed number of edges is significant. The pathway analysis and the interaction network helped generate a potential mechanism leading to cardiac impairment in adulthood from PGR (Figure 7).

### 2.8. Echocardiography

Echocardiography results obtained during adulthood are displayed in Table 2. Left ventricular (LV) mass was significantly reduced by 12.3% in PGR males and 10.3% in PGR females as compared to CON males and females, respectively (*p* = 0.03). Stroke volume (*p* = 0.01) and cardiac output (*p* = 0.02) were significantly reduced in the PGR group compared to the CON group. Myocardial performance index [(IVCT + IVRT)/LVET], also referred to as Tei index, was significantly increased (*p* = 0.02) in the PGR group compared to the CON group, which may represent impaired ventricular compliance.

A significant Diet x Sex interaction was seen during aortic ejection time (AET) (*p* = 0.042) and mitral valve A-wave (MV A) (*p* = 0.044), with CON females having the longest duration when compared to other groups. This resulted in a statistically different (*p* = 0.045) MV E/A ratio, where the CON females and PGR males had a lower ratio than the CON males and PGR females.

Additionally, the PGR group had a longer isovolumetric contraction (*p* = 0.02) and relaxation (*p* = 0.03) time compared to the CON group.

## 3. Discussion

The developmental origins of health and disease (DOHaD) hypothesis states growth restriction increases the risk for chronic disease in adulthood [6]. Specifically, low birth weight and low infantile weight at 1-year lead to a 47% increased risk for hypertension and CVD in adulthood [2,5]. The World Health Organization approximates that 161 million children a year have a restricted nutritive environment in utero and/or in postnatal life. Growth restriction has been clinically defined as two standard deviations below the length-for-age/height-for-age World Health Organization Child Growth Standards median.

Using diet manipulation, the present investigation showed that body mass was reduced until PN80 for PGR mice, which weighed significantly less than controls (Figure 1). Absolute heart mass of PGR mice was significantly less than controls at PN21, but not at PN80, suggesting heart mass was recuperated even though body mass was reduced (Figure 2). These findings support previous growth restriction investigations [16,17], which demonstrate preferential organ development for the heart and brain. The “preferential sparing” of vital organs may come at the expense of peripheral structures, such as skeletal muscle and bone, leading to reduced body mass [3].

While heart mass was recovered from PN21–80, functional impairments existed into adulthood. The PGR group had impairments in global cardiac function and mass (LV mass, stroke volume, cardiac output, myocardial performance index [MPI]), and diminished diastolic/systolic function (prolonged isovolumetric contraction/relaxation) in adulthood. These findings support the existing DOHaD literature, which has shown that PGR in mice impairs functional capacity of the heart, calcium flux, and exercise capacity, leading to an increased risk for CVD and mortality in adulthood [12,15,17,23,24,28,29,30,31,32,33,34,35,36,37].

Currently, there is limited information on the molecular mechanisms associated with cardiac impairment from growth restriction. From the existing literature, protein kinase B (*Akt*) and endothelin-1 (*Edn1*) are shown to be associated with CVD caused by early life growth restriction [34,38,39]. The present investigation did not identify differences in protein abundance of those previously identified proteins, possibly due to differences in methodology [4,6,7,16,20,24,29,40,41,42,43,44]. Studies showing *Akt* activation used placental arterial ligation to reduce nourishment to the fetus [39,45], and Neerhof et al. showed that *Edn1* is activated through platelet-activating factors in the ischemic intrauterine environment [39]. Thus, this indicates that mechanisms for PGR and intrauterine growth restriction may differ.

The mice in this investigation were genetically homogeneous, thus any change in protein abundance is due to the 21-day window of PGR. PN1–21 is associated with cardiomyocyte endowment, attainment of LV mass [23], and increased contractile strength [46,47,48,49]. As such, the proteomic screening was implemented immediately following the nutritional deficit, comparing CON vs. PGR hearts. Then, REACTOME revealed pathways (Figure 5) in the areas of extracellular matrix organization/muscle contraction and cell cycling, while the interaction networks from STRING (Figure 6A–D) showed significant PPIs from five nodes. These results relate to existing literature showing impaired cardiomyocyte nucleation [7,24,30,50,51] and cardiac muscle contraction/cell structure [46,47,52,53,54,55,56] following growth restriction. From this robust analysis, we propose a potential mechanism (Figure 7) whereby PGR alters the extracellular matrix and cardiac cell cycle, which may influence the CV impairment seen in adulthood.

### Impaired Extracellular Matrix, Cardiac Cell Cycle

From 2D DIGE, the PGR mice showed a significant reduction in collagen abundance at PN21 and again at PN80, when compared to the CON mice. Collagen abundance is necessary for proper extracellular matrix organization, cardiac structure, and functional durability in adulthood [54]. Col1a1 and Col3a1 are used as markers for valvular integrity [56], cardiac fibrosis [55], and connective tissue disease [53]. In this study, PGR mouse hearts showed prolonged isovolumetric contraction and relaxation times, indicating myocardial fibrosis. Additionally, reduced protein abundance of Col1a1 and Col3a1 using transgenic mice has previously shown cardiac impairment [57]. Therefore, PGR in mice potentially disrupts the abundance of Col1a1 fibers, which may lead to impaired myocardial function and an altered extracellular matrix in adulthood.

At PN21, 2D DIGE (*p* = 0.019) and western blotting (*p* = 0.007) showed that p57^kip2^ abundance was significantly less in the PGR mice compared to CON mice. Cyclin-dependent kinase inhibitors play a role in cardiac cell development by regulating proliferation [50,51,58]. Haley et al. demonstrated that p57^kip2^ knockout mice undergo increased cellular apoptosis in the heart [59] with altered cellular proliferation [60]. The alterations in p57^kip2^ at PN21 may be responsible for the impaired cardiomyocyte binucleation from growth restriction in early life, as shown previously [15,17,21,23,24,61,62]. However, it is worth mentioning that our experiments were conducted using whole heart lysates, thus we cannot rule out the role that p57^kip2^ may be playing in non-cardiomyocytes (fibroblasts, endothelial cells, vascular smooth muscle cells, etc.). The reduced abundance of p57^kip2^ also thins the trabecular layer of the developing heart, reducing mass and contractile force (Figure 7) [63]. Ferguson et al. previously showed reductions in binucleation and cell cycle activity at PN80 in PGR hearts, however, the present investigation demonstrated p57^kip2^ is not different in adulthood [17]. Thus, early life protein abundance differences have lasting effects on adult biology.

From 2D DIGE, the PGR mice displayed a reduced Ttn abundance at PN21, which was not significant at PN80 when compared to the CON mice. Ttn binds to myosin and operates as a framework for the thick myofilament [64], regulating cardiac muscle force production [65,66]. Radke et al. showed that *N2b* deletion on the Ttn gene leads to cardiac dysfunction and cardiac atrophy [47], similar to the pathology seen in our PGR mice (Table 2; reduced LV mass).

## 4. Materials and Methods

All experiments were conducted in accordance with the Guide for the Care and Use of Laboratory Animals and were approved by the Institutional Animal Care and Use Committee at Michigan State University (project identification code: 10/15-152-00, date of approval: 8 October 2015). Animals were housed in a climate-controlled vivarium, in a single room maintained at 21 °C with a 12 h light/dark cycle, on wood-chip bedding, and provided food ad libitum.

### 4.1. Nutritive Model

**Figure 8:**
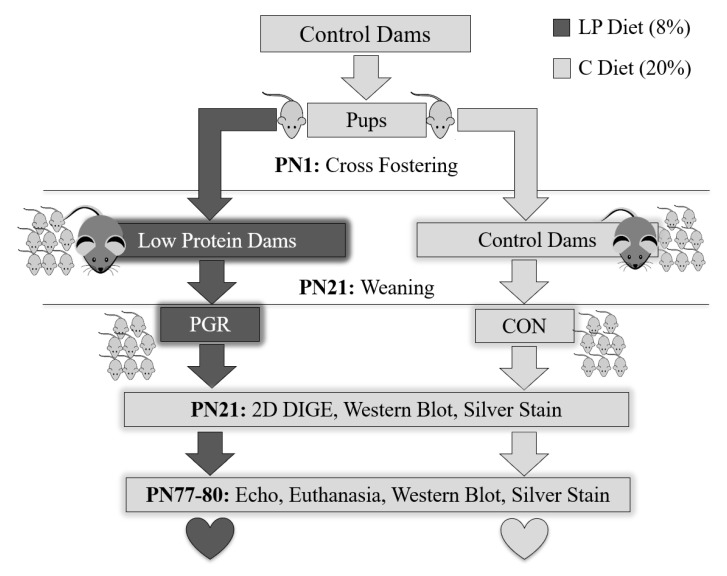
Experimental design. Two weeks prior to mating, dams were fed either a control diet (C: 20% protein) or isocaloric low-protein diet (LP: 8% protein). At birth, pups born to LP dams were sacrificed, while CON pups were cross-fostered to either a different control-fed dam (CON group) or a low-protein-fed dam (PGR group). At PN21, mice were weaned, a sub-cohort was euthanized, and hearts collected for 2D DIGE, western blotting, and silver nitrate staining (CON; *n* = 3 males; *n* = 3 females and PGR; *n* = 3 males; *n* = 3 females). PGR was restricted to the developmental window of PN1–21 as the remaining mice were fed the C diet from PN21–PN80. At PN77, cardiac function was evaluated by echocardiography (CON; *n* = 4 males; *n* = 3 females and PGR; *n* = 4 males, *n* = 5 females). Mice were given 2 days of recovery, then at PN80 were euthanized and hearts extracted for western blotting and silver nitrate staining.

PGR was induced using diet manipulation [20] (Figure 8). Briefly, third parity Friend leukemia virus B (FVB) (Charles River Laboratories, Wilmington, MA, USA) dams were fed a semi-purified control diet (20% protein; Research Diets, New Brunswick, NJ, USA) based on AIN93G, or a low-protein (LP; 8% protein) isocaloric diet two weeks prior to mating (the composition of the diets has previously been described [12]). To ensure all pups were born at the same time, breeding was conducted by placing one male in a female cage for only 24 h. At postnatal day (PN) 1, all pups born to females fed the LP diet were euthanized. Pups born to control-fed dams were combined and distributed to either: (1) Control (CON) group: pups born to a control dam then cross-fostered to a different control dam or (2) Postnatal Growth Restriction (PGR) group: pups born to a control dam then cross-fostered to an LP-fed dam. Dams that consume an LP diet produce 15–20% less milk which also has fewer free amino acids and a higher fatty acid content compared to controls, resulting in pups having an 18% reduction in caloric consumption, thus experiencing PGR [67,68,69,70,71,72,73].

All experimental litters were standardized to equal size, sex ratio, and body mass. Each dam received eight pups (four males, four females) and individual pups within a litter were given a tattoo identification. Litter size was maintained during lactation with the addition of donor pups of comparable age to replace any natural deaths; donor pups were not studied. At PN21, a sub-cohort (CON; *n* = 3 males, *n* = 3 females and PGR; *n* = 3 males, *n* = 3 females) was placed under 1% isoflurane anesthesia and euthanized via cervical dislocation. Hearts were extracted and then analyzed by 2D DIGE, then proteins were confirmed with western blot or silver nitrate staining. The remaining littermates (CON; *n* = 4 males; *n* = 3 females and PGR; *n* = 4 males, *n* = 5 females) were weaned, and fed the control diet until PN80. Thus, growth restriction was restricted to a developmental window (PN1–PN21) of postnatal life.

Body mass was measured bi-weekly from PN1–PN28, and then weekly from PN28–PN80 using a calibrated small animal weighing scale (Ohaus Corporation, Parsippany, NJ, USA; CS Series). At PN77, cardiac function was analyzed via echocardiography and Doppler analysis. Mice recovered for 2 days, then at PN80 were euthanized as described above. Hearts were removed, weighed, and stored in a −80 °C freezer for western blotting and silver nitrate staining to determine if proteomic differences persisted or were recovered in adulthood. Heart mass is reported as absolute and standardized to body surface area, using Meeh’s formula [74]:

Body surface area = 9.662 × (body weight) ^0.667^ (1)

The standardization of cardiac variables to body surface area allows for the comparison of cardiac function between mice of different sizes [74,75]. Tibia length was measured as a surrogate for body composition and lean tissue growth using digital Vernier calipers (General Tools, Secaucus, NJ, USA) [76].

### 4.2. Two-Dimensional Differential In-Gel Electrophoresis (2D DIGE)

2D DIGE and protein identification with MS followed procedures previously described [18,19,77,78]. Protein was extracted from PN21 hearts of CON and PGR mice using liquid nitrogen pulverization with a mortar and pestle then dissolved in DIGE labeling buffer (7 M urea, 2 M thiourea, 4% 3-Cholamidopropyl dimethylammonio 1-propanesulfonate (CHAPS), 30 mM Tris, pH 8.5). Extracted proteins were quantified using Bradford reagents (Pierce Chemical Company, Dallas, TX) with a bovine serum albumin (BSA) standard. Samples were randomized and fluorescently labeled by reacting 40 µg of cardiac protein and 200 pmol of either Cy3 or Cy5 CyDye DIGE Fluors (GE Healthcare, Chicago, IL). One Cy3- and one Cy5-labeled sample were loaded on a single gel, along with the Cy2-labeled pooled samples of CON and PGR. The pooled, Cy2-labeled samples act as an internal normalization standard, allowing each protein spot to be semi-quantitatively compared within each gel and amongst all gels. Proteins were iso-electrically focused on 24 cm Immobiline Dry Strips (IPG DryStrips: pH 4–7; GE Healthcare) using an IPGPhor (GE Healthcare) with the following protocol: 500 V for 1 h followed by a linear gradient to 1000 V over the span of one hour. A linear gradient to 8000 V until a total of ~60,000 volt∙hour was reached. The focused strips were equilibrated in two steps: (1) 15 min in SDS equilibration buffer I (6 M urea, 2% SDS, 30% glycerol, 50 mM Tris, pH 8.8, 0.01% bromophenol blue, and 10 mg·mL^−1^ Dithiothreitol (DTT)) followed by (2) 15 min with equilibration buffer II in which the DTT was substituted by 25 mg·mL^−1^ iodoacetamide. The equilibrated IPG strips were positioned on top of 12% polyacrylamide SDS slab gels and covered with 1% low-melt agarose and run in a DALT 6 system (GE Healthcare; 10 °C) at 1 W per gel until the dye front reached the bottom of the gels [79].

**Figure 9:**
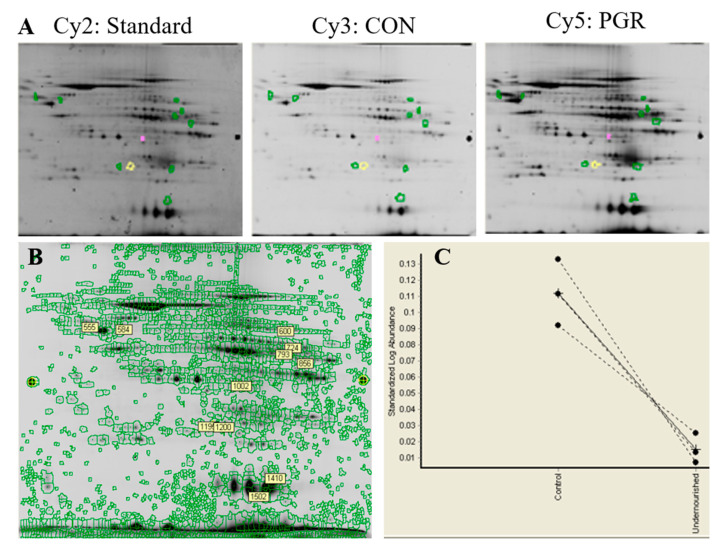
Representative image from DeCyder software: (**A**) Six gels were CyDye-labeled through 2D DIGE three-dye methodology. (**B**) Of the 2187 total spots identified, two spots were determined to have significant differential abundance (*p* < 0.05); three spots with a *p*-value of 0.05–0.075 were also robotically picked and digested, illustrated by the green, yellow, and pink circles in panel A. (**C**) The graphical view from the DeCyder software shows the standardized log abundance between the CON and PGR of the protein spot that was selected.

Multiplexed gel images were acquired using a Typhoon Trio (GE Healthcare), and viewed using ImageQuant software (GE Healthcare, version 8.1). Images were loaded into DeCyder software (GE Healthcare, version 6.5) and differences in protein abundance (*p* < 0.05), along with the magnitude of abundance (average ratio), were quantified (Figure 9). Average ratio was derived from the normalized spot volume standardized against the internal standard, thus providing the degree of abundant change between identified proteins. Gels used for spot picking were fixed in 10% methanol and 7.5% acetic acid overnight.

Spots displaying significant changes (*p* < 0.05) in abundance between PGR and CON mice were robotically picked and digested (Ettan Picker and Digestor; GE Healthcare) with recombinant porcine trypsin from the gels (Promega, Madison, WI, USA) as described previously [80]. Spots with a *p*-value greater than 0.05 but less than 0.075 were also picked and identified (Table 1), to provide potential future hypothesis-driven investigations. Extracted tryptic peptides were dried by Speed-Vac, sequenced, and identified by nano-LC/MS/MS on an LTQ XL (Thermo-Finnigan, San Jose, CA, USA) using the MASCOT and X! Tandem search engines. The MASCOT program (v2.2) searched the *Mus musculus* proteome in the NCBInr database using the following limitations and allowances for protein identification: (1) one missed cleavage by trypsin; (2) monoisotopic peptide masses; (3) peptide mass tolerance of 1.2 Da; (4) fragment mass tolerance of 0.8 Da. Additionally, oxidation of methionine (variable modification) and carbamidomethylation (fixed modification) of cysteine were allowed by MASCOT in the protein identification. Proteins were required to have a minimum of three matching peptides to form the identification. Protein identifications were then verified by Scaffold (Proteome Software, Portland, OR, USA).

### 4.3. Western Blotting

Cdkn1c (p57^kip2^) was identified as a protein of interest by 2D DIGE (see results) and abundance was confirmed through western blotting at PN21, and again at PN80 to determine if the protein signature persisted into adulthood. Extracted protein from 2D DIGE was incubated in LaemmLi buffer [81] and separated using a self-prepared 9% acrylamide gel via SDS-PAGE. Proteins were then transferred to polyvinylidene difluoride (PVDF) membranes (VWR, Radnor, PA, USA) and blocked for 1 h in proteomic grade non-fat dried milk. Primary antibodies were used with the manufacturer’s recommendations (Cell Signaling, Danvers, MA; Gapdh Rabbit *mAb*, 1:1000; and Abcam, Cambridge, UK; p57^kip2^, ab75974), then standardized to Gapdh (a.u.; arbitrary units) to ensure equal loading of proteins on SDS-PAGE. Membranes were incubated in a secondary horseradish peroxidase antibody (Abcam, Cambridge, UK; Goat Anti-Rabbit IgG HRP, ab6721) in a 1:2500 ratio for 1 h. Development and detection of blots was completed using a Kodak Image Station 2000R (Kodak, Hempstead, UK). Blots were quantified by densitometry software (Carestream Molecular Imaging, Woodbridge, CT, USA).

### 4.4. Silver Nitrate Staining

In addition to Cdkn1c (p57^kip2^), titin (Ttn) and collagen fibers (Col1a1, Col3a1, and Col4a5) were identified as proteins of interest (see results) via 2D DIGE and were confirmed using silver nitrate staining of SDS-PAGE.

For confirmation of the relative change in abundance between samples of Ttn and collagen proteins, the protocol by Zhu et al. for SDS gel electrophoresis was used, as western blotting does not yield reliable results for Ttn [82,83] and collagen due to reduced specificity of antibody binding [83,84,85]. PGR and CON samples from 2D DIGE were prepared with protease inhibitors (Roche, Basel Switzerland; cOmplete Protease Inhibitor Cocktail) in LaemmLi buffer and heated at 45 °C for 7 min. CON or PGR protein (2 µg) were loaded into the wells of a hand-cast, 6% SDS-polyacrylamide gel for electrophoretic separation at 30 mA for 2.5 h. Throughout electrophoresis, gels were chilled with a water coolant system maintained at 2–10 °C. Following electrophoresis, gels were silver stained following the procedure set forth previously by Blum et al. [86]. Gels were then imaged and inspected for high molecular weight proteins (140–245 kDa) [87]. Bands between Col1a1 (140–210 kDa) and Col3a1 (110–140 kDa) were determined based on molecular weight. Images were then loaded into ImageJ software for quantification by densitometry software (U.S. National Institutes of Health, Bethesda, MD, USA).

### 4.5. Generation of a Potential Mechanism by which PGR Influences Cardiac Impairment

Following 2D DIGE and a secondary confirmatory technique, potential metabolic pathways in which the proteins might be involved were generated using REACTOME [88,89] and STRING [90,91,92,93] software. As previous genetic and proteomic literature has used REACTOME and STRING together for the development of pathological disease mechanisms [94,95], corresponding gene codes for the proteins were entered into REACTOME to determine pathways related to the identified proteins from PGR. The following functions within REACTOME were used: (1) Description: summarizes the protein in the pathway browser, such as input/output molecules, catalysts, regulators, and references with supporting evidence; (2) Structure: details the 3D structure from the Protein Data Bank; (3) Gene expression: expression information from the Gene Expression Atlas (Cambridgeshire, UK). The software then analyzes all of the identified pathways through (a) entity p-value: the probability that the overlap between the query and the pathway has occurred by chance, (b) entities false discovery rate: probability corrected for multiple comparisons, and (c) reaction ratios: the total reactions in the pathway divided by the total number of reactions for the entire species tissue selected (heart).

STRING (version 10.5) was used to obtain a network of protein–protein interactions (PPIs) and has been used in previous proteomic research [90,91,92,96]. The database uses five different sources to quantitatively integrate known and predicted PPIs including genomic context predictions, high-throughput lab experiments, conserved co-expression, text-mining, and previous knowledge in databases. An adjustable modification to only allow the highest confidence score was made (0.900) in the STRING settings, thus reducing the number of false discoveries. The network allows for understanding functional activities of the proteins identified from 2D DIGE [97].

### 4.6. Echocardiography

Structural and functional parameters of the heart as a result of PGR were assessed by echocardiography (Vevo 770 ultrasound, with 30 MHz transducer, Visualsonics, Toronto, Canada) in adulthood as previously described [98]. At PN77, mice were maintained under 1% isoflurane anesthesia on a heated board with their limbs restrained. Measurements were performed in 2D and M modes with images taken in the short axis at the level of the papillary muscles and used to determine LV systolic and diastolic dimensions. Mitral and aortic blood flow velocities were measured by Doppler from the apical view (10 MHz pulsed Doppler probe with real-time Doppler spectrum analyzer, Indus Instruments, Webster, TX, USA). Recordings were saved for offline analysis and were completed with the observer blinded to the diet group.

### 4.7. Statistics

A linear model compared growth curves between the CON and PGR groups (JMP V12.0 Sass, Cary, NC). Two-way ANOVAs were used with the main effects of diet (CON vs. PGR) and sex (male vs. female) to compare heart mass (absolute or standardized), tibia length, and western blot protein abundance. An ANCOVA was used to analyze echocardiography parameters with heart rate and body surface areas as covariates. An α level of 0.05 was set a priori and, if necessary, Tukey’s honestly significant different (HSD) post hoc test was used for multiple comparisons. The 2D DIGE statistical differences (*p* < 0.05) in spot intensity used DeCyder software as previously described [77,78]. Briefly, numerical data for individual spots detected through DeCyder software are automatically calculated and compared based on the volume (sum of pixel intensity), area (spot radius covered), peak height (pixel value at the X, Y position of the spot), and slope of the protein spot (gradient associated with the 3D attributes of a spot map pair). REACTOME and STRING software both had α levels set a priori at 0.05 to reduce false discovery rates.

## 5. Conclusions

PGR during the first 21 days of mouse life reveals proteomic differences in the heart compared to controls. From 2D DIGE, these alterations in protein abundance center on the structural impairments to the extracellular matrix (Col1a1/Col3a1), and regulation of the cardiac cell cycle (p57^kip2^). These proteomic alterations in the PGR mouse hearts were associated with cardiac impairment (reduced mass and indicators of fibrosis) in adulthood as evaluated by echocardiography and Doppler analysis. Future directions should aim to modulate the expression of these identified proteins to mitigate cardiac impairment.

### Limitations and Future Directions

Due to limited sample volume from PN21 mouse hearts, the present investigation only confirmed proteins based on the existing literature. Future growth restriction research using proteomics should concentrate on the other significantly identified proteins from 2D DIGE. Due to the imprecise reliability of western blotting for Ttn and a reduced specificity of antibodies for collagen proteins, silver nitrate staining was used to confirm the relative protein abundance between our PGR and CON mouse hearts [46,47,82,83,84,85,99]. The authors acknowledge that Ttn and collagen are not the only proteins found in the reported high molecular weight range, but our 2D DIGE results combined with nano-LC/MS/MS and echocardiography results allow us to consider Ttn and collagen as proteins of interest. Lastly, further work should focus on identifying proteins in the pH 4–7 region, as proteins outside of this isoelectric focus were not targeted through 2D DIGE.

## Figures and Tables

**Table 1:** Differential protein abundance between control (CON) and growth-restricted (PGR) mice.

Spot Number	Spot *p*-Value	Average Ratio of Spot	Protein	No. of Identified Peptides	Expressed in (CON/PGR)	Category
1200	0.0042	−1.22	Peroxiredoxin 6 (Prdx6)	6	CON	Metabolism, Cellular Signaling
600	0.019	−1.2	Titin (Ttn)	4	CON	Muscle Organization
600	0.019	−1.2	Rho GTPase-Activating Protein 29 (Arhga29)	4	CON	Signal Transduction
600	0.019	−1.2	Cyclin-Dependent Kinase Inhibitor 1C-p57^kip2^ (Cdkn1c)	3	CON	Cell Cycle
600	0.019	−1.2	SAFB-like Transcription Modulator (Sltm)	3	CON	Metabolism
600	0.019	−1.2	Exportin 5 (Xpo5)	3	CON	Cell Cycle, Signal Transduction
600	0.019	−1.2	Crumbs Cell Polarity Complex Component (Patj)	3	CON	Cell–Cell Communication
600	0.019	−1.2	Collagen Type I α 1 (Col1a1)	3	CON	Extracellular Matrix
600	0.019	−1.2	Histone-Lysine N-Methyltransferase (Setd2)	9	CON	Gene Expression (Transcription)
600	0.019	−1.2	Collagen Type IV α 5 (Col4a5)	5	CON	Extracellular Matrix
600	0.019	−1.2	Collagen Type III α 1 (Col3a1)	9	CON	Extracellular Matrix
600	0.019	−1.2	Dynein Axonemal Heavy Chain 1 (Dnah1)	4	CON	Protein Transportation (Motor)
856	0.064	−1.26	Aldolase, Fructose-Bisphosphate A (Aldoa)	20	CON	Metabolism (Fatty Acid Oxidation)
856	0.064	−1.26	Acetyl-CoA Acetyltransferase 1 (Acat1)	17	CON	Metabolism (Fatty Acid Oxidation)
856	0.064	−1.26	Acetyl-CoA Acyltransferase 2 (Acaa2)	13	CON	Metabolism (Fatty Acid Oxidation)
856	0.064	−1.26	Aspartate Aminotransferase (Got1)	12	CON	Metabolism (Biosynthesis Proteins)
856	0.064	−1.26	Microtubule-Associated Protein 6 (Map6)	6	CON	Extracellular Matrix
856	0.064	−1.26	Leucine-Rich Repeats and Ig-like Domains 3 (Lrig3)	5	CON	Signal Transduction
856	0.064	−1.26	Acyl-CoA Dehydrogenase Medium Chain (Acadm)	4	CON	Metabolism (Fatty Acid Oxidation)
856	0.064	−1.26	Aconitase 2 (Aco2)	5	CON	Metabolism (Krebs Cycle)
584	0.075	1.34	Caveolae-Associated Protein 1 (Ptrf)	11	PGR	Gene Expression (Transcription)
584	0.075	1.34	Vitamin D-Binding Protein (Gc)	14	PGR	Metabolism, Protein Transportation
584	0.075	1.34	Neuroblast Differentiation-Associated Protein (Ahnak)	3	PGR	Structural Protein, Cardiac Ca^2+^ regulation
584	0.075	1.34	Heat Shock Protein Family D (Hspd1)	4	PGR	Metabolism, Signal Transduction, Cell Cycle
584	0.075	1.34	Vimentin (Vim)	4	PGR	Programmed Cell Death, Muscle Contraction
584	0.075	1.34	Adenomatous Polyposis Coli (Apc)	4	PGR	Metabolism, Signal Transduction
584	0.075	1.34	ATP Synthase F1 Subunit β (Atp5b)	4	PGR	Metabolism (Ox. Phos.)
584	0.075	1.34	Potassium Calcium-Activated Channel Subfamily M α1 (Kcnma1)	4	PGR	Intracellular Ion Regulation
1410	0.075	1.34	Fetuin-A (Ahsg)	5	PGR	Immune System
1410	0.075	1.34	Hemoglobin Subunit β (Hbb)	3	PGR	Transport of Small Molecules

Spot Number, the spot that was robotically picked from gels; *p*-Value, significance of the differential protein abundance from DeCyder software; Average Ratio, originated from the normalized spot volume normalized alongside the intra-gel standard provided by DeCyder software analysis, offering a measure of the abundance variances between proteins detected; Protein (gene code), name of identified protein with the associated gene that codes for the protein; No. of Identified Peptides, the number of peptides recognized from the selected gel spot; Abundant in (CON/PGR), the diet group in which the protein is over-expressed; Physiological Category, the functional pathway with which the identified protein is associated.

**Table 2:** Echocardiography between control (CON) and growth-restricted (PGR) mice.

Parameter	CON		PGR		*p*-Value		
	Males	Females	Males	Females	Diet	Sex	Diet*Sex
Global Cardiac Function and Mass							
Heart Rate (beats·min^−1^)	404.5 ± 29.0	391.3 ± 41.0	365.0 ± 29.0	362.6 ± 25.9	NS	NS	NS
Area, S (mm^2^)	12.1 ± 1.1	10.7 ± 1.4	9.4 ± 0.9	9.4 ± 0.9	NS	NS	NS
Area, D (mm^2^)	18.7 ± 1.0	16.6 ± 1.5	14.3 ± 1.0	13.6 ± 0.9	NS	NS	NS
LV Mass (mg)	115.7 ± 3.5 **^A^**	111.5 ± 2.3 **^A^**	101.4 ± 1.3 **^B^**	100.0 ± 2.6 **^B^**	**0.03**	NS	NS
Stroke Volume (µL)	45.7 ± 2.5 **^A^**	43.2 ± 2.8 **^A^**	38.9 ± 2.4 **^B^**	36.8 ± 2.1 **^B^**	**0.01**	NS	NS
Cardiac Output (mL·min^−1^)	18.5 ± 1.3 **^A^**	16.9 ± 1.8 **^A^**	14.2 ± 1.3 **^B^**	13.4 ± 1.2 **^B^**	**0.02**	NS	NS
Myocardial Performance Index	0.49 ± 0.03 **^A^**	0.43 ± 0.04 **^A^**	0.59 ± 0.03 **^B^**	0.55 ± 0.03 **^B^**	**0.02**	NS	NS
LV Mass Corrected (mg)	92.5 ± 2.0 **^A^**	89.3 ± 1.2 **^A^**	81.1 ± 1.8 **^B^**	80.0 ± 1.2 **^B^**	**0.04**	NS	NS
Diastolic and Systolic Function							
Ejection Fraction (%)	66.2 ± 4.5	67.3 ± 6.2	62.0 ± 4.4	61.7 ± 3.9	NS	NS	NS
Fractional Shortening (%)	36.9 ± 3.3	37.8 ± 4.6	34.2 ± 3.2	33.2 ± 2.9	NS	NS	NS
LVID, S (mm)	2.7 ± 0.2	2.4 ± 0.2	2.8 ± 0.2	2.9 ± 0.2	NS	NS	NS
LVID, D (mm)	4.0 ± 0.2	3.8 ± 0.3	4.2 ± 0.2	4.1 ± 0.2	NS	NS	NS
AET (ms)	52.3 ± 1.6 **^A^**	59.2 ± 2.2 **^B^**	51.4 ± 1.6 **^A^**	53.2 ± 1.4 **^A^**	-	-	**0.042**
MVDT (ms)	26.0 ± 4.6	22.0 ± 6.3	21.1 ± 4.4	21.2 ± 4	NS	NS	NS
MV A (mm/s^2^)	320.4 ± 30.3 **^A^**	448.1 ± 41.6 **^B^**	335.2 ± 29.5 **^A^**	328.6 ± 27.0 **^A^**	-	-	**0.044**
MV E (mm/s^2^)	598.7 ± 46.0	675.1 ± 63.2	523.4 ± 44.9	641.1 ± 40.4	NS	NS	NS
MV E/A	1.9 ± 0.2 **^A^**	1.5 ± 0.2 **^B^**	1.6 ± 0.2 **^B^**	2.0 ± 0.1 **^A^**	-	-	**0.045**
IVCT (ms)	10.8 ± 1.4 **^A^**	8.6 ± 1.9 **^A^**	12.7 ± 1.4 **^B^**	11.9 ± 1.3 **^B^**	0.02	NS	NS
IVRT (ms)	15.1 ± 1.1 **^A^**	16.9 ± 1.5 **^A^**	18.0 ± 1.1 **^B^**	17.5 ± 0.9 **^B^**	0.03	NS	NS

LV: left ventricle. S: systole. D: diastole. LVID, S: left ventricle internal diameter (systole). LVID, D: left ventricle internal diameter (diastole). AET: ejection time from opening to closing of the aortic valve. MVDT: deceleration time. MV A: blood flow velocity following atrial contraction. MV E: blood flow velocity during early diastole. MV E/A: ratio of MV E to MV A. IVCT: left ventricle isovolumetric contraction time. IVRT: isovolumetric relaxation time. Values are means ± standard error. Difference in superscript letters across a row indicates statistical significance for post hoc analysis. Bold *p*-values indicate statistical significance.
